# Multidisciplinary Home-Based Rehabilitation Program for Individuals With Disabilities: Longitudinal Observational Study

**DOI:** 10.2196/59915

**Published:** 2024-10-16

**Authors:** Patricio Barría, Asterio Andrade, Daniel Gomez-Vargas, Alejandro Yelincic, Flavio Roberti, Eduardo Bahamonde, Rolando Aguilar, Bessie Cordova

**Affiliations:** 1 Rehabilitation Center Club de Leones Cruz del Sur Punta Arenas Chile; 2 Department of Electrical Engineering University of Magallanes Punta Arenas Chile; 3 Institute of Automatics National University of San Juan San Juan Argentina

**Keywords:** rehabilitation, home-based therapy, physical therapy, psychological therapy, home physiotherapy, disabilities, occupational therapy, personalized care, patient care, motor disorder, mood disorder, motor function

## Abstract

**Background:**

Disability affects a significant portion of the global population nowadays, necessitating innovative approaches to access rehabilitation processes. Home-based rehabilitation has emerged as a beneficial approach, offering comfort and context-specific therapy.

**Objective:**

This study aims to evaluate the impact of a multidisciplinary home-based rehabilitation program for individuals with moderate neuromusculoskeletal disabilities in terms of motor function and mood.

**Methods:**

A total of 270 participants with median age of 66 (IQR 20-98) years were recruited from the National Disability Registry of Chile. The intervention involved a multidisciplinary team composed of 49 health care professionals providing personalized treatment plans over 4 months (32 sessions for physical therapy, 8 sessions for occupational therapy, 4 sessions for nutrition, 8 sessions for psychology, and 4 sessions for nursing and podiatry). This program also included 2 medical evaluations (at the beginning and the end) to monitor clinical progress in terms of motor function and mental health, using the Berg Balance Scale and Beck Depression Inventory, respectively.

**Results:**

The home-based rehabilitation program showed significant improvements (*P*<.001) in motor function and balance with a reduction in fall risk. Specifically, the Berg Balance Scale score decreased close to 15% after the home-based rehabilitation program for all enrolled participants. On the other hand, depression levels showed no significant changes (*P*=.27), with percentages of variation less than 8% between the 2 assessed conditions. In this sense, participants remained with the same mild depression level (14 of 63) concerning the Beck Depression Inventory score.

**Conclusions:**

This study concludes that personalized home-based rehabilitation programs are effective in enhancing motor function and balance, particularly in individuals with neurological conditions. On the other hand, the findings in terms of mood advocate for further exploration of psychological support within such programs to enhance overall patient well-being.

**Trial Registration:**

ClinicalTrials.gov NCT06537791; https://clinicaltrials.gov/study/NCT06537791

## Introduction

Disability is a complex and multidimensional phenomenon that affects approximately 16% of the global population, equivalent to around 1.3 billion people [1]. According to the World Health Organization, disability results from the interaction between individuals’ health conditions and different personal and environmental factors, such as negative attitudes and barriers related to transportation and access to public buildings. The Centers for Disease Control and Prevention also identifies disability as a condition that hinders the performance of fundamental activities and interactions with the world, affecting vital aspects such as movement and thinking [2].

In this sense, home-based rehabilitation has emerged as an innovative and growing response, providing health services in the patient’s home rather than in a hospital or medical institution. Home-based rehabilitation is not only more comfortable for patients and their families, but it can also be more effective than rehabilitation in a hospital setting, as it allows patients to receive therapy in a realistic and specific context for their situation [3,4]. However, the home-based implementation presents unique challenges, such as care coordination among multiple health care providers and the need to ensure access to necessary resources for home rehabilitation [5].

Home-based rehabilitation has gained relevance due to the reported evidence related to effectiveness and user preferences. Specifically, a comparative study between hospital-based and this personalized approach revealed a distinct preference among both patients and staff for the home-based method, owing to its tailored and goal-oriented therapeutic strategies [3]. Furthermore, previous studies have also reported the benefits in patients for home-based therapies compared with hospital-based methods, considering the home environment is more conducive to adaptation and realism [4].

The home-based therapy’s relevance could particularly extend to physiotherapy for patients with neurological diseases. These home-based programs could offer ongoing therapy opportunities, benefiting the retention of intervention effects and showing improvements in mobility, muscle strength, and balance [6]. Saggini et al [7] reported significant improvements in autonomy, motor skills, and quality of life during home-based rehabilitation for patients with chronic stroke. This study also emphasized the importance of a familiar and personalized environment in the rehabilitation process of the involved patients [7].

On the other hand, home-based methodologies, being more personalized and patient-centered, reflect a significant shift in how we approach health care and well-being. Thus, the home-based concept can align with the modern vision of rehabilitation as a crucial strategy for enhancing individuals’ capacity to carry out daily activities and participate in society [8]. Notwithstanding, despite the advances and promising results in home-based strategies, it is necessary to recognize the limitations and gaps in current research, highlighting the need for further rigorous studies to evaluate the effectiveness and cost-effectiveness of these programs [6].

In this context, the aim of this study is to evaluate the functioning of a comprehensive home-based rehabilitation program for individuals with moderate neuromusculoskeletal-origin disabilities in the Magallanes region (Chile). The proposed program aims to offer continuous and tailored care within the familiar environment of the patients, emphasizing the pivotal role of home-based rehabilitation in promoting optimal outcomes.

## Methods

### Recruitment

This study enrolled 270 people with moderate neuromusculoskeletal disabilities who were officially registered on the National Disability Registry of Chile. This protocol targeted individuals of all ages, provided they received referrals from recognized medical institutions in the Magallanes region. These institutions included the “Dr. Lautaro Navarro Avaria” Clinical Hospital, the “Dr. Augusto Essmann Burgos” Hospital in Puerto Natales, the “Marcos Chamorro I.” Hospital in Puerto Porvenir, as well as primary health care facilities.

**Figure 1:**
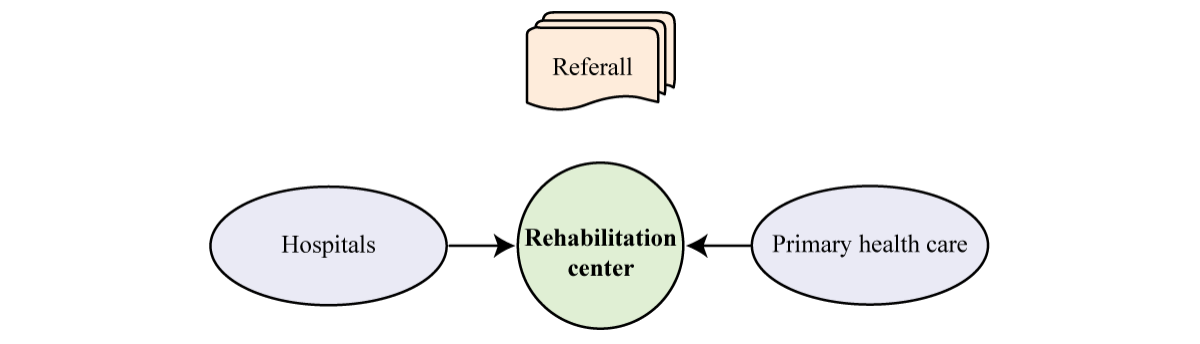
Enrollment diagram implemented in this study.

The inclusion criteria covered participants with medical referrals and clinical information available in the rehabilitation center's database (Figure 1). This requirement ensured that participants had a documented medical history accessible for a more accurate evaluation and monitoring of their health status. The exclusion criteria encompassed individuals with severe cognitive impairments that hinder their ability to follow the rehabilitation program, participants with acute medical conditions who require immediate hospitalization, and pregnant women.

### Intervention

The home-based rehabilitation program implemented a comprehensive approach supported by a multidisciplinary team of 49 health care professionals. Participants received 4 months of a personalized treatment plan to ensure comprehensive and coordinated care.

Regarding therapies, physiotherapists provided 32 sessions per patient, focused on physical rehabilitation, while occupational therapists offered 8 sessions to improve independence in daily activities. Nutritionists conducted 4 sessions to optimize nutritional intake, and psychologists provided 4 sessions of emotional and cognitive support. In addition, this study provided four nursing and podiatry sessions focused on general health care or foot care.

Overall, the rehabilitation program provided approximately 82 hours of therapy to each patient, encompassing all clinical interventions. Specifically, participants received around 20.5 hours of therapy per month, which implies 5.12 hours per week, with each session lasting 60 minutes. This collaborative and personalized approach aimed to ensure coverage of various areas of health and well-being, as well as the effectiveness of treatment in the home setting, promoting comprehensive and maintained recovery of the patients.

This study also included two medical assessments, one at the beginning (ie, baseline) and one at the end (ie, home-based program) of the study, to monitor clinical progress and adjust treatment as necessary. In parallel, social workers conducted intake and discharge assessments to address the social and environmental patients’ needs.

### Study Outcomes

#### Patients’ Characteristics and Health Conditions

The clinical and demographic characteristics of the patients were recorded and analyzed, evaluating their health status and level of functional improvement before and after their participation in the rehabilitation program. This approach allowed for a detailed understanding of the program’s impact on the target population.

#### Motor Function

Considering the consequences of falls in patients with neuromusculoskeletal disabilities, the home-based rehabilitation program aimed to improve participants’ balance during the sessions. In this sense, medical staff measured the Berg Balance Scale using a questionnaire in the 2 medical assessments. The Berg Balance Scale estimates balance capacity through a scoring system ranging from 0 (inability to maintain balance independently) to 56 (independent balance). From this range, lower scores (ie, below 45) indicate a potential necessity for assistance to mitigate falls and ensure patient safety.

#### Mental Health

In the mental health realm, addressing depression is fundamental, particularly within the context of home-based rehabilitation programs. Thus, this study included an assessment using the Beck Depression Inventory-II (BDI) at the beginning and end of the proposed intervention. BDI is a revised tool for assessing the severity of depression, using a 21-item scale that classifies symptoms into 4 levels. Scores of 0 to 13 indicate minimal depression, suggesting mild or nonexistent symptoms. Scores of 14 to 19 describe mild depression, where symptoms are more noticeable but still manageable. A range between 20 and 28 points denotes moderate depression, with symptoms that may significantly interfere with daily life. Finally, scores of 29 to 63 represent severe depression, with intense symptoms that generally require immediate clinical intervention.

### Statistical Analysis

This study conducted a descriptive analysis to explore the enrolled patients’ demographics, focusing on age, gender, and diagnosis distribution. In assessing the age distribution, mean and SD were used to estimate the central tendency and dispersion of the participants’ ages. Furthermore, age data were segmented into percentiles and quartiles to understand age distribution patterns within the sample. In addition, this study quantified the number and proportion of participants based on gender and diagnosis, providing a comprehensive overview of the demographic landscape.

On the other hand, a Shapiro-Wilk test was applied to determine the normality of the variable distributions. In this sense, it is possible to define which statistical tests are appropriate for data analysis. Significant deviation from normality was considered for Shapiro-Wilk *P* values less than .05. Thus, data that did not follow a normal distribution used nonparametric statistical methods such as the Wilcoxon rank sum test. For the Wilcoxon test, statistical significance in the analyzed parameters was set at *P*<.05.

The calculations and statistical analyses carried out in this study were accomplished using Python (version 3.11.5; Python Software Foundation) and Pandas (version 2.1.3; Pandas Development Team). This software provides support for data processing, analysis, and graphing.

### Ethical Considerations

The study was approved by the institutional review committee of the Rehabilitation Center Club de Leones Cruz del Sur (approval code CRCS_UID_010223), ensuring compliance with ethical and methodological standards. All data were treated confidentially and anonymized. No compensation was provided to participants for their involvement in this study.

## Results

### Patients’ Characteristics and Health Conditions

**Figure 2:**
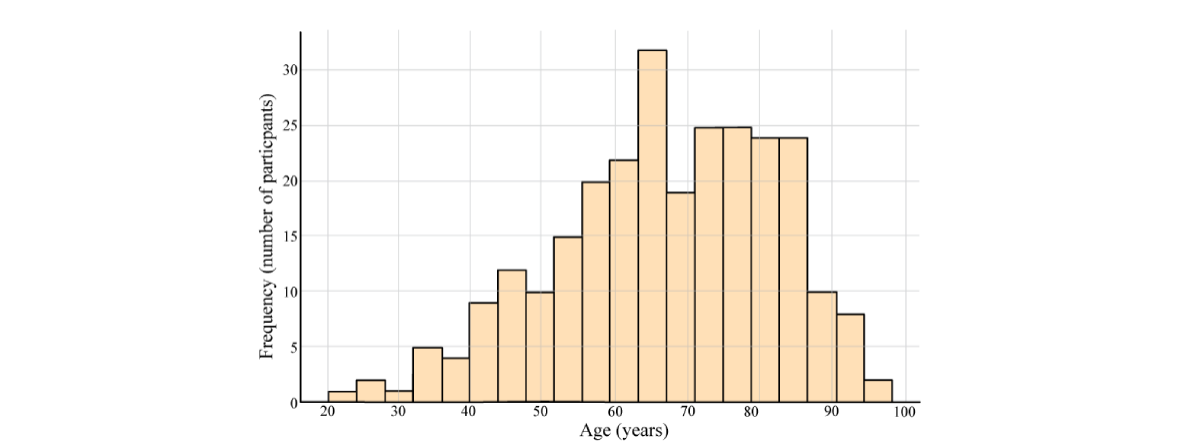
Histogram of age distribution of participants.

This study analyzed a sample of 270 patients for the motor function outcomes and 187 for the psychological health. The complete sample exhibited a wide variability in the participants’ age, reflecting a broad distribution within an adult population. The mean age was 66.7 (15.3) years, indicating a tendency toward an older age group as shown in Figure 2. Likewise, the participants’ age range was extensive, with a minimum of 20 years and a maximum of 98 years (Figure 2), exhibiting considerable variability in this study.

Regarding participant gender, the selected sample reflected an uneven distribution. Specifically, out of the 270 participants analyzed, 198 (73.3%) were female and 26.7% (72/270) of them were male.

Considering the inclusion criteria, the study sample was classified into 2 main diagnostic categories: (1) musculoskeletal diseases and (2) neurological diseases. The first category comprehended most cases, with 188 representing 69.6% of the analyzed sample. On the other hand, the neurological diseases category involved 82 participants, constituting 30.4% of the group.

For the musculoskeletal diseases category, prevalent diagnoses reported by the participants encompassed osteoarthritis and fibromyalgia. These conditions typically manifest as pain and dysfunction within patients’ joints and muscles, often constituting primary motivations for consultation and treatment within this classification. In the neurological diseases category, Parkinson’s disease and sequelae of cerebrovascular diseases emerged as the most prevalent diagnoses. These conditions typically impact both the nervous system and the motor capacity of affected individuals.

### Motor Function

#### Overall Sample

**Figure 3:**
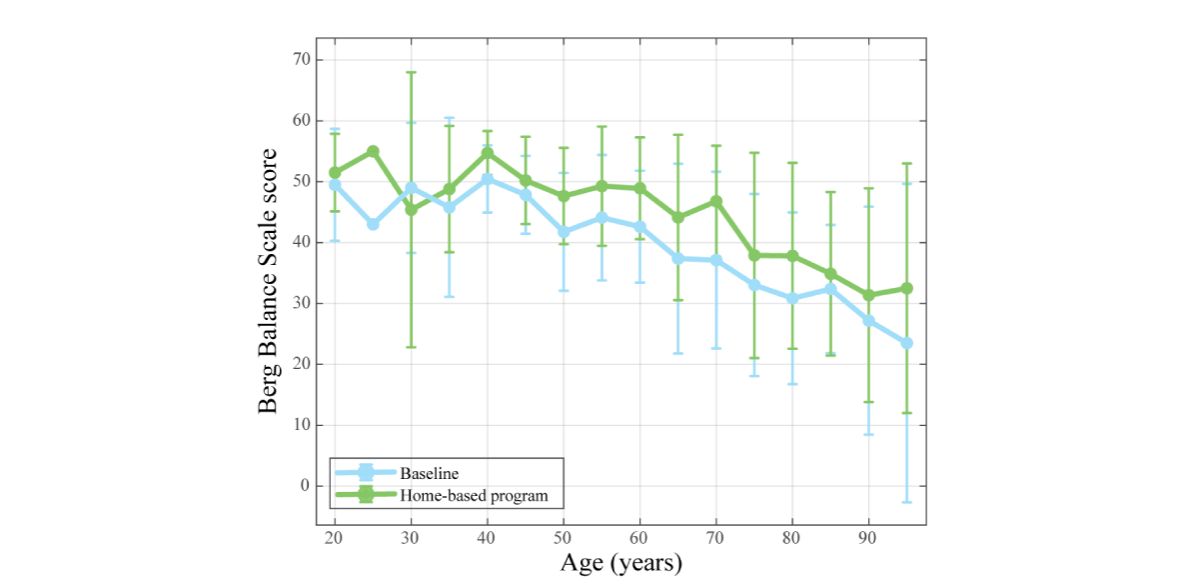
Mean (SD) of the Berg Balance Scale score across the age spectrum for the baseline and home-based program.

Regarding the motor function, this study presents comparative results of the scores on the Berg Balance Scale at 2 different time points, at the beginning (ie, baseline) and at the end (ie, home-based program). Figure 3 illustrates the mean value and variation of the Berg Balance Scale score across the age spectrum involved in this study. Overall, the Berg Balance Scale score increased for all age groups following the completion of the home-based program, except for the 30-year age group, which exhibited a slight decrease.

The data normality was verified through the Shapiro-Wilk test. The baseline condition did not follow a normal distribution (*P*<.001), so the Wilcoxon rank sum test compared the datasets. The statistical test revealed a significant difference between the baseline and home-based program (*P*<.001) for the participants’ balance measured from the Berg Balance Scale.

**Figure 4:**
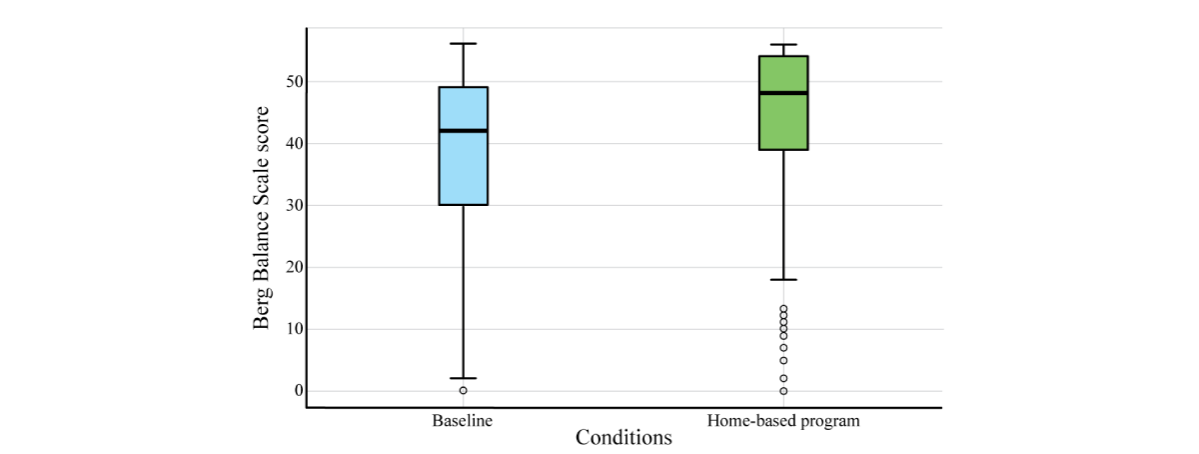
Distribution plot of Berg Balance Scale scores for the baseline and home-based program.

Figure 4 shows the variation between both assessed conditions (ie, baseline and home-based program), with a notable increase in the median and mean values (ie, 14.8% and 16.6%, respectively) and a slight reduction in the dispersion for the home-based program (ie, –3.5%).

A detailed breakdown of the means, medians, and SDs of the Berg Balance Scale scores at both time points highlights that at baseline, the mean score was 38.3 (SD 14.1) with a median of 42.0 (IQR 30.0-49.0), while after the home-based program, the mean increased to 44.0 (SD 13.6) with a median of 49.0 (IQR 39.0-54.0). Thus, this study exhibited a percentage variation between both conditions of 14.9% for the mean and 16.7% for the median.

#### Diagnostic Categories

**Figure 5:**
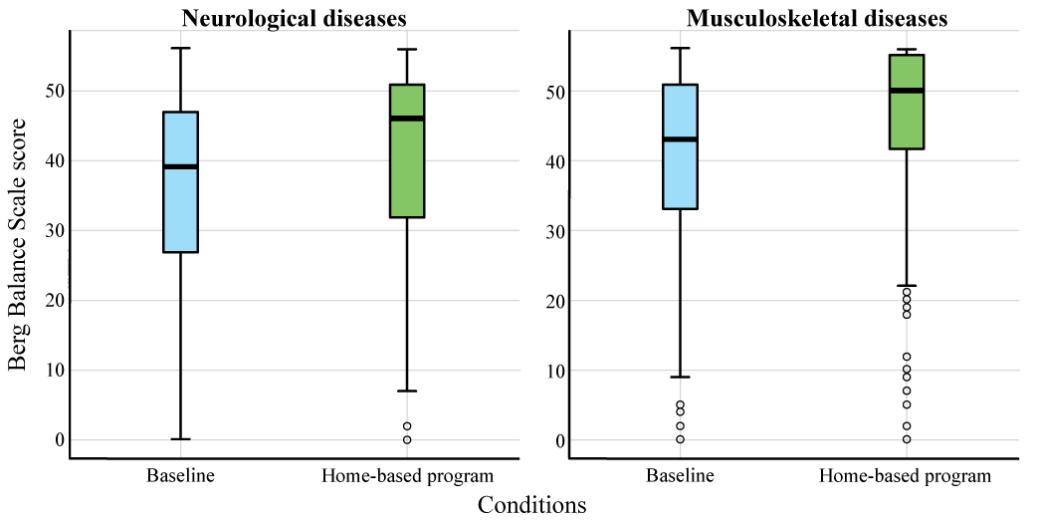
Distribution plot of Berg Balance Scale scores for neurological and musculoskeletal diseases.

These results present the score and statistical values for each divided category (ie, neurological diseases and musculoskeletal diseases), focusing on examining the differences between the baseline and the home-based program. Initially, the Shapiro-Wilk test demonstrated that all cases did not follow a normal distribution (*P*<.001). Therefore, the Wilcoxon rank sum test was carried out for each category. The Berg Balance Scale scores exhibited significant differences between the baseline and the home-based program for both categories, ie, *P*<.001 for neurological diseases and *P*<.001 for musculoskeletal diseases. Likewise, the tendency to increase the mean value and reduce dispersion is also exhibited in both categories (Figure 5) as the complete sample described in the previous section.

Table 1 compares the descriptive statistics, providing a detailed insight into the differences in each category for both assessed conditions (ie, baseline and home-based program).

**Table 1:** Descriptive statistics of Berg Balance Scale scores in both assessed conditions.

Conditions			Mean (SD)		Median (IQR)
**Neurological diseases**					
	Baseline	34.8 (15.1)		39.0 (27.0-47.0)	
	Home-based program	40.8 (14.6)		46.0 (32.0-51.0)	
	Variation (%)	17.2 (–3.3)		17.9 (18.5-8.5)	
**Musculoskeletal diseases**					
	Baseline	39.8 (13.4)		43.0 (33.0-51.0)	
	Home-based program	45.3 (12.9)		50.0 (41.0-55.0)	
	Variation (%)	13.8 (–3.7)		16.3 (24.2-7.8)	

### Mental Health

**Figure 6:**
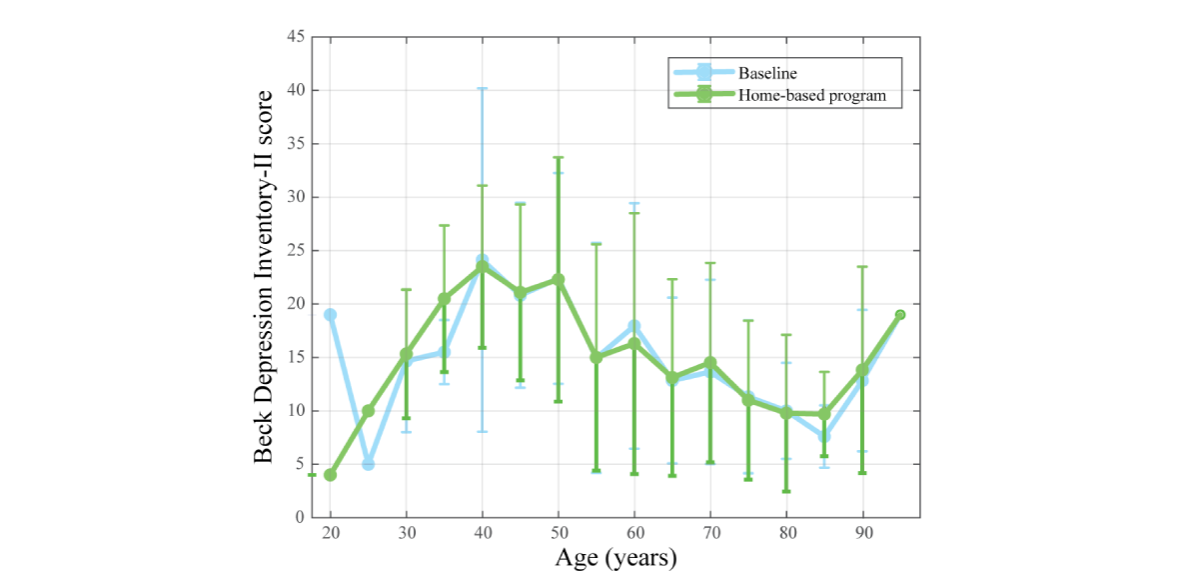
Mean (SD) of the Beck Depression Inventory-II score across the age spectrum for the baseline and home-based program.

This study focused on assessing depression levels using the BDI to measure the participants’ mental health within the evaluated condition (ie, baseline and home-based program). Considering the participants’ age spectrum, Figure 6 illustrates the BDI scores measured at the beginning and the end of this study for a subset of 187 participants from the total sample of 270. Overall, the BDI scores remained similar after completing the home-based program compared with the baseline.

**Figure 7:**
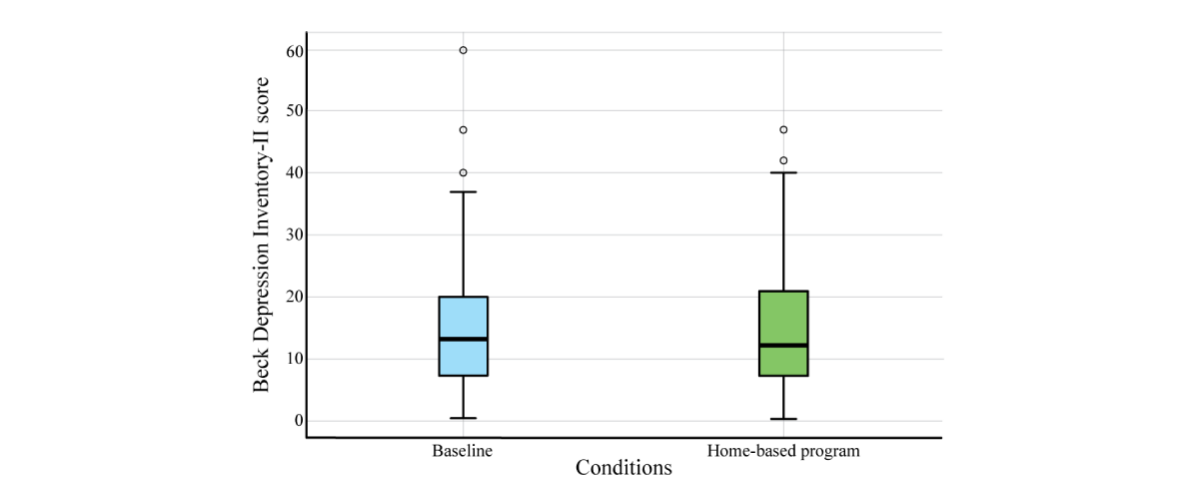
Distribution of Beck Depression Inventory scores for the baseline and home-based program.

In statistical terms, the Shapiro-Wilk test verified the data normality, finding that the home-based program did not follow a normal distribution (*P*<.001). In this sense, the comparison between conditions was accomplished with the Wilcoxon rank sum test. This test yielded a statistic of 2532.5 and a *P* value of around .27, so the BDI scores did not exhibit significant differences (*P*>.05) during the experiment. In the same line, Figure 7 illustrates the score distribution for both conditions, where medians and the IQR remain similar.

At baseline, the mean BDI score was 14.5 (SD 9.5) with a median of 13.0 (IQR 7.0-20.0). After the home-based program, the mean slightly increased to 14.6 (SD 9.7) with a median of 12.0 (IQR 7.0-21.0). The variation between both conditions shows a change of 0.7% for the mean and –7.7% for the median. Despite these changes, the participants remained within the same mild depression category (ie, BDI score of 14 to 19) after completing the rehabilitation program.

## Discussion

### Improvement of Motor Function

This study aimed to evaluate the effectiveness of a home rehabilitation program in improving motor function in participants. The results of the study indicated significant improvements in motor function as assessed by the Berg Balance Scale. In addition, these improvements were consistent across all age groups of participants. Statistical tests revealed statistically significant differences in scores between baseline and home-based program assessments, indicating improved balance and motor function. These findings highlight the effectiveness of the home rehabilitation program, which involved a multidisciplinary team of health care professionals providing personalized care. The comprehensive approach of the program, which encompassed medical assessments, social evaluations, and various therapeutic interventions, contributed to the observed improvements in motor function.

The program’s commitment to providing approximately 82 hours of therapy per patient over 4 months ensured that participants received intensive and individualized care. This approach addressed various aspects of health and well-being, resulting in substantial improvements in motor function. The dosage of therapy in rehabilitation is a crucial aspect of achieving effective outcomes in patients. Parameters such as frequency, intensity, duration, and timing of therapy must be carefully considered. Studies have shown that appropriate dosing can significantly improve recovery outcomes, while insufficient or excessive dosing can be less effective or even counterproductive. Therefore, rehabilitation professionals need to personalize the therapy dosage according to the individual needs of each patient [9].

These findings highlight the importance of adapting rehabilitation interventions to the specific needs and conditions of individuals. By understanding the unique challenges and potential for improvement in different patient populations, health care professionals can develop specific strategies to optimize motor function outcomes. The results of this study emphasize the need for comprehensive and personalized rehabilitation programs that address specific motor disabilities associated with both musculoskeletal and neurological conditions. This will help maximize the effectiveness of rehabilitation interventions and improve overall motor function and quality of life for individuals with these conditions.

The findings of the study have important implications for rehabilitation practice. They demonstrate the impact of a comprehensive and personalized approach to rehabilitation, especially in a home setting. The positive changes observed in motor function underline the importance of interventions and rehabilitation programs tailored to the specific needs of individuals with neurological conditions and those with musculoskeletal conditions. Personalized rehabilitation programs, especially those including exercises adapted to individual needs, have shown to be beneficial in improving physical outcomes in older adults living in the community. According to a systematic study conducted by Guichen et al [10], personalized exercise programs based on assessments of physical function can be a safe and effective approach to improving aspects such as balance, strength, mobility, physical activity, and disease symptoms in this population. Although the study revealed that these programs did not show advantages in terms of exercise adherence or economic benefits, the findings underscore the importance of considering both physical functions and psychological factors when developing personalized exercise programs for older adults. This research highlights the need for more high-quality studies with larger samples to better understand the effectiveness and attitudes of older individuals toward these personalized exercises [10].

In addition, the study found that participants in the musculoskeletal group had higher initial scores on the Berg Balance Scale than the neurological group. This suggests that individuals with musculoskeletal conditions may have better initial motor function than those with neurological conditions. The Berg Balance Scale proved to be an effective tool in assessing balance in patients with various musculoskeletal conditions. A key study in this area is by Bogle Thorbahn and Newton [11], where the use of the Berg Balance Scale in patients with different musculoskeletal conditions was explored [12]. The results indicated that the Berg Balance Scale is a valid and reliable instrument for measuring static and dynamic balance in this population. This finding is significant as balance is a crucial factor in the quality of life of patients with musculoskeletal conditions, directly influencing their ability to perform daily activities and reducing the risk of falls. Furthermore, the Berg Balance Scale provides a quantitative assessment that can be used to monitor patient progress over time and modify treatment plans accordingly.

Our study found that individuals with neurological conditions experienced greater improvement in motor function when participating in a home-based rehabilitation program compared with those with musculoskeletal conditions. These findings align with a recent study by Lim et al [12], which also found significant improvements in balance and gait in patients with chronic hemiparesis following a stroke who participated in a home-based rehabilitation program. These results highlight the importance of tailoring interventions and rehabilitation programs to address the specific needs of each patient group. In addition, despite lower initial scores in the neurological group, this group showed greater improvement in motor function compared with the musculoskeletal group. This indicates that the home-based rehabilitation program was particularly effective in addressing motor disabilities associated with neurological conditions [12].

Furthermore, the observed improvement in motor function in both the neurological and musculoskeletal categories on the Berg Balance Scale has important implications for fall risk. The Berg Balance Scale is a widely used tool for assessing balance and mobility, including items specifically assessing fall risk [11]. According to the categories defined by the Berg Balance Scale, individuals with lower scores are considered to have a higher risk of falls. In our study, the initial scores for both the neurological and musculoskeletal categories were within the medium fall risk range (21-40 points). However, the improvements observed in both groups indicate a reduction in fall risk. For the neurological category, the increase in mean scores from 34.76 to 40.84 suggests a shift from medium fall risk to low fall risk. Similarly, in the musculoskeletal category, the increase in mean scores from 39.84 to 45.34 reflects a movement from medium fall risk to low fall risk. These improvements in motor function and balance likely contribute to a decrease in fall risk in both categories.

It is important to note that the Berg Balance Scale is just one tool used to assess fall risk, and other factors such as muscle strength, gait, and cognitive function also play a role in determining an individual’s fall risk. However, the significant improvements observed in motor function in the neurological and musculoskeletal categories suggest a positive impact on reducing fall risk. Overall, the findings of this study highlight the potential of home-based rehabilitation programs to improve motor function and reduce fall risk in individuals with neurological conditions and those with musculoskeletal conditions. By targeting specific disabilities and providing personalized care, these programs can contribute to better balance and mobility, ultimately leading to a decrease in fall risk and an overall improvement in the quality of life for individuals with these conditions.

Scientific evidence indicates that physical rehabilitation interventions can significantly decrease the risk of falls in patients with disabilities. A systematic study found that in patients with knee osteoarthritis, physical therapies such as strength training and aerobic exercises notably improved balance and reduced the risk of falls [13]. In addition, other research revealed that exercise interventions decrease the number of falls by 32% and the number of individuals experiencing falls by 22% among healthy older adults, underscoring the value of posture-challenging exercises in fall prevention [14]. These studies demonstrate the effectiveness of physical rehabilitation interventions in reducing fall risk, which is crucial for improving safety and quality of life in patients with disabilities and vulnerable populations.

### Mental Health Analysis

The results for patients on the BDI indicate that, on average, they presented mild levels of depressive symptoms both in the initial and final assessments. In addition, this trend was consistent across the different age groups involved in the study. These findings could be related to the substantial proportion of women involved in this study, particularly in relation to depressive symptoms. Specifically, previous studies have reported gender differences in depression prevalence and response to therapeutic interventions, with women often experiencing higher rates of depression than men [15,16]. The predominance of women in our sample could have contributed to the overall pattern of mild depressive symptoms observed, potentially reflecting gender-specific factors that are not fully addressed by this study.

On the other hand, the relationship between musculoskeletal and neurological disabilities and depressive symptomatology has been the subject of research in various studies. For example, a study published by Chimenti et al [17] addresses how rheumatological diseases such as rheumatoid arthritis and spondyloarthritis may be strongly associated with the development of alterations in the cognitive behavioral sphere, particularly with the development of depression. This association is attributed to various factors, including increased pain, fatigue, reduced health-related quality of life, increased levels of physical disability, and higher health care costs. In addition, the possible role of proinflammatory cytokines in the development of central nervous system manifestations is explored, suggesting a link between inflammation and depressive symptoms in these conditions [17].

In another study conducted by Yalew et al [18], the magnitude of depression and associated risk factors were specifically examined in patients with musculoskeletal disorders treated in an outpatient physiotherapy department. A significant prevalence of potential depression was found among patients, with 57.1% of patients showing signs of potential depression. This study also investigated factors such as treatment duration, social support, and pain intensity, and how these related to the prevalence of depression in patients with musculoskeletal disorders [18].

Furthermore, several studies explore the relationship between neurological diseases and depressive symptomatology. One specific study focused on finding an association between neurological disorders and symptoms of anxiety and depression in a vulnerable population. In this study, a significant relationship was found between various neurological pathologies and anxious and depressive symptoms. Disorders such as cerebrovascular diseases and epilepsy showed higher severity in these symptoms compared with other disorders such as headaches. Around 112 assessed patients had high scores on a psychological distress scale, indicating a high risk of developing anxiety and depression disorders. This study underscores the importance of a comprehensive assessment of patients with neurological disorders to identify and treat possible symptoms of anxiety and depression [19]. These studies highlight the importance of considering the psychological and emotional implications in patients with musculoskeletal and neurological disabilities and suggest the need for a comprehensive approach that addresses both physical symptoms and associated mood disorders.

Our analysis of scores on the BDI did not reveal statistically significant differences between initial and control assessments. This result suggests that the participants’ mood remained relatively stable throughout the program despite receiving psychological sessions. This suggests that although participants received fewer sessions of psychological and emotional support, the program may have contributed to the stability of the participants’ mood. Regarding the optimal therapeutic dose of therapy, Bruijniks et al [20] explored the effectiveness of cognitive-behavioral therapy and interpersonal therapy with different frequencies for treating depression. It is suggested that twice-weekly sessions may be more effective and lead to faster recovery from depressive symptoms than once-weekly sessions. This study suggests that a limited number of sessions, such as the 8 received by patients in our study, may not be sufficient to achieve significant changes in the mood of patients with depression.

### Limitations

Although this study provides significant results and important implications, it also has several limitations that should be considered. First, the study only included participants who were referred and able to participate in a home-based rehabilitation program, which may introduce selection biases and sample heterogeneity. In addition, the study relied on self-reported measures, such as the BDI, which may be subject to social desirability biases. Furthermore, the study did not include a control group receiving standard care or compare the home-based rehabilitation program with other rehabilitation interventions, limiting the ability to determine the specific effects of the program. Finally, the study only assessed motor function and mood and did not explore other important outcomes such as quality of life or activities of daily living.

### Prospects and Next Steps

Our study highlights the effectiveness of a home-based rehabilitation program in improving motor function, emphasizing the importance of a multidisciplinary and holistic approach to rehabilitation. The need for personalized care and intensive therapy to achieve better outcomes is emphasized. Future research should focus on evaluating the long-term effects of these programs, as well as exploring additional measures to assess improvements in motor function. It also proposes investigating the potential benefits of increasing the frequency or intensity of psychological and emotional support sessions within the program, which could further enhance the well-being of participants and reduce depressive symptoms. These suggestions aim at optimizing the rehabilitation program, considering both the physical and emotional aspects of recovery. Future research should also address the limitations described in the previous section and further investigate the effectiveness of home-based rehabilitation programs in larger and more diverse populations, using objective measures and comparing different rehabilitation approaches.

### Conclusions

In conclusion, this study provides significant evidence that a home-based rehabilitation program is effective in improving motor function in individuals with neurological conditions and those with musculoskeletal conditions. The results demonstrate that participants experienced notable improvements in motor function and a reduction in the risk of falls, especially in the group with neurological conditions. This finding highlights the importance of a multidisciplinary and comprehensive approach to rehabilitation, encompassing both the physical and emotional aspects of recovery. Although no significant differences were found in depression levels, the study suggests the possibility of further enhancing these programs by intensifying sessions of psychological and emotional support. In summary, the study reinforces the relevance of personalized care and intensive therapy in rehabilitation and invites future research to evaluate the long-term effects of such programs and explore additional measures for a more comprehensive assessment of improvements in motor function.

## Abbreviations

BDI: Beck Depression Inventory-II

## Data Availability

Data are available on reasonable request by email to PB (pbarria@rehabilitamos.org).
